# Plasma levels of apolipoprotein-E in residents of the European North of Russia

**DOI:** 10.1186/1476-511X-12-43

**Published:** 2013-03-27

**Authors:** Anastasiya M Kaneva, Evgeny R Bojko, Natalya N Potolitsyna, Jon O Odland

**Affiliations:** 1 Institute of Physiology, Komi Science Center, Ural Branch of Russian Academy of Sciences, Pervomaiskaya av. 50, Syktyvkar, 167982, Russia; 2 Faculty of Health Sciences, University of Tromso, Tromso, Norge, NO-9037, Norway

**Keywords:** Apolipoprotein-E, Lipid metabolism, Age, The North, Adaptation

## Abstract

**Background:**

Apolipoprotein-E (apoE) is one of the metabolically active apoproteins and plays an important role in lipid metabolism. However, there are no data on levels of apoE in residents of the North in spite of the fact that specific features of lipid metabolism in the northerners are described. The present work was designed to study plasma levels of apoE in residents of the European North of Russia.

**Methods:**

A total of 937 native residents of the European North of Russia (463 men and 474 women**)** aged 13–60 years were included in the study. ApoE concentrations in the blood plasma were measured by immunoturbidimetric method.

**Results:**

Plasma levels of apoE in residents of the European North of Russia were low. ApoE concentrations below the defined normal values were detected in 57.0% of the men and in 59.2% of the women. The mean plasma levels of apoE did not significantly differ in men and women (2.80 mg/dl vs 2.87 mg/dl). Plasma apoE concentrations in residents of the European North of Russia changed with age. Plasma levels of apoE decreased from 13 to 21 years in men and from 13 to 35 years in women and then increased in both sexes (p < 0.001).

**Conclusion:**

The limits of variation of plasma apoE levels in residents of the European North of Russia shift towards lower values. Plasma levels of apoE below normal values were observed in approximately half of investigation subjects.

## Background

Apolipoprotein-E (apoE) is a monomeric glycoprotein with 299 amino acid residues and 34 kDa molecular mass [1]. The protein is a structural and functional constituent of plasma chylomicrons and very low density lipoproteins (VLDL) and their lipolytic degradation products (i.e. chylomicron remnants and intermediate density lipoproteins). ApoE is also found in some subfractions of high density lipoproteins (HDL) [2-4]. This apolipoprotein is synthesized in liver and extrahepatic tissues (brain, kidneys, adrenal glands, spleen, muscles, skin, macrophages, etc.). Plasma apoE is largely liver-derived [5,6].

ApoE plays important role in lipid metabolism. The main function of apoE is to regulate uptake of lipoproteins from the circulation by receptor-mediated endocytosis [4,7]. ApoE also participates in the synthesis of some lipoproteins, in the transport and redistribution of lipids among various tissues including the cholesterol transport from peripheral tissues in the liver, in the reparation of nervous cells and the vascular wall. Moreover, this protein has been shown *in vitro* to have antioxidant and anti-inflammatory properties [5,8-16].

Considerable influence of apoE on the lipid profile has been shown in studies on apoE-deficient mice and transgenic rabbits expressing high levels of apoE. ApoE-deficient mice have extremely high lipid levels and massive amounts of cholesterol-rich *β*-VLDL, resulting in development of severe atherosclerotic lesions even on a normal diet [17-19]. On the other hand, high plasma levels of apoE may also be associated with increased risk of dyslipidemia. It is supposed that high levels of apoE increase atherosclerosis risk by stimulating hepatic VLDL production and inhibiting VLDL lipolysis. Thus there is an optimal range of plasma apoE levels that is maximally beneficial, and that levels above or below that range impose a risk rather than a benefit for atherosclerosis [5,20]. In normolipidemic subjects, normal values of plasma concentration of apoE are 3-7 mg/dl [4].

At present time, major attention is given to the studying of polymorphism of the аpоЕ gene. There are three common alleles of the apoE gene (ε2, ε3, ε4) coding three isoforms (E2, E3, E4). Population studies have shown that apoE isoforms influence lipid and apolipoprotein levels and are important determinants of risk for the development of cardiovascular diseases [21-24]. Plasma apoE concentration is usually not taken into account in similar studies. However, it is known that levels of apoE can change or even mask the apoE gene polymorphism effects on lipid parameters [25,26]. Hence studying the apoE gene polymorphism effects on plasma lipid levels without taking into account apoE concentration could lead to confounding results. Meanwhile, influence of the apoE gene polymorphism on apoE levels was not always found [27,28]. Besides, the ε3 allele in all populations is the most frequent, with a range of 67-80% [29]. So we consider that the intent attention to the apoE gene polymorphism and insufficient interest in levels of this protein might not be correct.

The data on biological variations of plasma apoE levels are contradictory [3,25,30-36]. There are not much data on concentration and influence of apoE on lipid profile in residents of the North in spite of the fact that specific features of lipid metabolism in the northerners were described [37,38].

## Methods

### Subjects and sampling

Subjects were apparently healthy residents of the European North of Russia (natives from five areas of Komi Republic located between 62° and 65° North latitude, 68% of Russians and 32% of Komi). The total number of subjects participating was 937; consisting of 463 men and 474 women aged between 13-60 years. The subjects of both sexes were divided into the following age groups: 13-15 years (71 boys and 80 girls), 17-21 years (97 men and 101 women), 22-35 years (101 men and 107 women), 36-45 years (90 men and 85 women), and 46-60 years (104 men and 101 women).

Participants were excluded from the study by the following criteria: (i) a body mass index (BMI) of 30 or greater (BMI = weight in kilograms divided by height in meters squared); (ii) total cholesterol concentration above 5.5 mmol/l; (iii) triglycerides concentration above 1.8 mmol/l; (iv) glucose concentration above 6.0 mmol/l. All participants were considered as being free from serious and chronic illnesses at the time of the recruitment. Each subject gave written informed consent for participating in the study, which was approved by the ethics committee of Institute of Physiology, Komi Science Center, Ural Branch of Russian Academy of Sciences.

A single blood sample of 5 ml was taken by rapid venipuncture with minimum stasis in the morning at 9 am after an overnight fast of 12-13 h. The samples were collected into vacutainers (Becton Dickinson BP). Blood samples were centrifuged and plasma was placed into eppendorf microcentrifuge tubes and was stored at -20°C until analysis.

### ApoE measurements

ApoE concentration in the blood plasma was measured by immunoturbidimetric method using a kit from «Chronolab» (Switzerland, Cat. No 101-0550). The samples were analyzed immediately after thawing at 37°C in thermostatic bath. Measurement of each sample was carried out in duplicate, and the mean was calculated. Absorbance of all samples was measured on the Power Wave-200 automated spectrophotometer (Bio-Tek Instruments, USA) at 340 nm.

### Analytical procedures

Total cholesterol, triglycerides, HDL-cholesterol and glucose concentrations in the blood plasma were determined using enzymatic methods on the Power Wave-200 automated spectrophotometer (Bio-Tek Instruments, USA) with commercially available kits («Chronolab», Switzerland).

### Statistical analysis

Statistical analysis was performed with Statistica 6.0 (Statsoft, Tulsa, USA). Continuous variables are presented as median and interquartile range (25th and 75th percentiles), and qualitative variables are expressed as relative frequencies. Continuous data were analyzed using the Mann-Whitney (for two groups) and the Kruskal-Wallis tests (for three and more groups). Where the Kruskal-Wallis test revealed significant effect, the Mann-Whitney test with Bonferroni correction for multiple comparisons was used to discern differences between groups. The chi-squared test was used for comparison of qualitative variables. Correlations between indices were assessed using the Spearman rank correlation. A value of p < 0.05 was accepted as statistically significant.

## Results

Table 1 gives the baseline characteristics of the study subjects. All subjects had BMI, lipids and glucose levels within the laboratory normal range by virtue of the exclusion criteria. These parameters increased with age in both men and women (p < 0.001).

**Table 1:** The baseline characteristics in residents of the European North of Russia

	**N**	**Body mass index, kg/m**^**2**^	**Total cholesterol, mmol/l**	**Triglycerides, mmol/l**	**HDL-cholesterol, mmol/l**	**Glucose, mmol/l**
**Men, n = 463**						
1 group	71	19.2	3.75	0.83	1.06	3.83
13-15 years		(17.4; 21.3)	(3.33; 4.20)	(0.69; 1.02)	(0.91; 1.27)	(3.38; 4.63)
2 group	97	19.4	3.55	0.93	0.99	3.99
17-21 years		(18.1; 20.9)	(2.63; 4.09)	(0.80; 1.10)	(0.86; 1.39)	(3.48: 4.60)
3 group	101	25.1	4.05	1.16	1.29	4.41
22-35 years		(23.1; 26.9)	(3.53; 4.56)	(0.92; 1.33)	(0.88; 1.51)	(4.04; 4.84)
4 group	90	25.4	4.46	1.13	1.35	4.49
36-45 years		(23.0; 27.5)	(3.89; 4.86)	(0.93; 1.36)	(1.16; 1.62)	(4.03; 4.88)
5 group	104	26.7	4.31	1.02	1.42	4.56
46-60 years		(24.3; 28.4)	(3.86; 4.75)	(0.86; 1.20)	(1.12; 1.62)	(4.09; 5.08)
*p value*		*<0.001*	*<0.001*	*<0.001*	*<0.001*	*<0.001*
**Women, n = 474**						
1 group	80	20.2	3.65	0.84	1.06	3.67
13-15 years		(18.4; 22.1)	(3.27; 4.26)	(0.70; 1.05)	(0.95; 1.30)	(3.17; 4.34)
2 group	101	20.1	3.59	0.94	1.13	3.86
17-21 years		(18.0; 22.1)	(3.11; 4.13)	(0.81; 1.07)	(0.96; 1.49)	(3.42; 4.27)
3 group	107	20.8	3.88	0.81	1.47	3.93
22-35 years		(19.1; 23.5)	(3.58; 4.33)	(0.69; 0.98)	(1.05; 1.79)	(3.43; 4.55)
4 group	85	23.9	4.29	0.87	1.60	4.13
36-45 years		(21.3; 27.6)	(3.84; 4.68)	(0.68; 1.08)	(1.13; 1.81)	(3.73; 4.74)
5 group	101	26.9	4.44	0.94	1.62	4.30
46-60 years		(25.4; 27.9)	(4.03; 4.83)	(0.80; 1.14)	(1.41; 1.88)	(3.92; 4.85)
*p value*		*<0.001*	*<0.001*	*<0.001*	*<0.001*	*<0.001*

Data are expressed as median (interquartile range).

*p value*; Statistical significance of differences between age groups was estimated using the Kruskal-Wallis test.

Plasma concentrations of apoE in residents of the European North of Russia are presented in Table 2. In residents of the European North of Russia, there were no significant differences in plasma levels of apoE between men and women (p = 0.652). The results showed that the mean plasma apoE levels were 2.80 mg/dl in men and 2.87 mg/dl in women (Table 2). The analysis of individual data has shown that apoE concentrations below normal values were observed in 57.0% of the men and 59.2% of the women. The maximum deviation of plasma apoE levels from the lower limit of normal values reached 78.3% in men and 86.7% in women. There were no subjects with plasma levels of apoE above normal values. No significant differences in percentage of subjects with low and normal values of apoE between men and women were detected (χ^2^ = 0.002; p = 0.962).

**Table 2:** Plasma apolipoprotein-E levels (mg/dl) in residents of the European North of Russia by sex and age

**Age group**	**Men**	**Women**	***p***^***1***^***value***
Total	2.80 (1.97; 3.72)	2.87 (2.07; 3.65)	*0.652*
13-60 years	n = 463	n = 474	
1 group	2.70 (1.91; 3.84)	2.96 (1.94; 3.68)	*0.886*
13-15 years	n = 71	n = 80	
2 group	2.19 (1.57; 3.00) ^*1-2^	2.52 (1.64; 3.29)	*0.147*
17-21 years	n = 97	n = 101	
3 group	2.35 (1.66; 3.08)	2.45 (1.87; 3.25)	*0.262*
22-35 years	n = 101	n = 107	
4 group	2.98 (2.23; 3.73) ^**2-4; 3-4^	3.15 (2.32; 3.86) ^**2-4; 3-4^	*0.309*
36-45 years	n = 90	n = 85	
5 group	3.50 (2.88; 4.07) ^*1-5; **2-5; 3-5^	3.11 (2.39; 3.95) ^**2-5; 3-5^	*0.094*
46-60 years	n = 104	n = 101	
*p*^*2*^*value*	*<0.001*	*<0.001*	

Data are expressed as median (interquartile range).

*p*^*1*^*value*; Statistical significance of differences between men and women was estimated using the Mann-Whitney test.

*p*^*2*^*value*; Statistical significance of differences between age groups was estimated using the Kruskal-Wallis test. Differences between age groups are statistically significant at: * - p < 0.05; ** - p < 0.01 (the Mann-Whitney test with Bonferroni correction).

Plasma levels of apoE in residents of the European North of Russia changed with age (Table 2). Plasma apoE concentrations were higher in the adolescent group (13-15 years) compared with subjects 17-35 years old. Men and women aged 17-21 years had apoE levels on 19% (p = 0.040) and 15% (p = 0.305) less than the adolescents. The significant increase of plasma apoE concentrations in residents of the European North of Russia was observed after age 35. The age-related increase of plasma apoE levels in men was simultaneously accompanied by significant decrease of number of subjects with apoE concentrations below normal values (χ2 = 17.07; p = 0.002) (Table 3). The lowest percent of subjects with apoE levels below normal values was observed in the group after 45 years. In women the number of subjects with low values of apoE also tended to decrease with age but these changes were statistically insignificant (χ2 = 7.01; р = 0.135).

**Table 3:** The percentage of subjects with values of apolipoprotein-E below normal values in the age groups of residents of the European North of Russia

**Age group**	**Men (%)**	**Women (%)**
Total 13-60 years	57.0	59.2
13-15 years	57.7	52.2
17-21 years	74.2	68.3
22-35 years	73.3	71.0
36-45 years	52.2	42.4
46-60 years	28.8	47.5
*p value*	*0.002*	*0.135*

Data are presented as percentage.

*p value*; Statistical significance of differences between age groups was determined by Chi-square test.

Strong influence of age on BMI, lipids and glucose levels (p < 0.001) was confirmed by means of the Spearman’s rank correlation (Table 4). Meanwhile, apoE was correlated only with total cholesterol (r_s_ = 0.24; p < 0.001) in men and with total cholesterol (r_s_ = 0.15; p < 0.001) and triglycerides (r_s_ = -0.10; p = 0.037) in women. No significant correlation between plasma apoE levels and BMI was observed. These data indicate that age-related changes of apoE concentrations in residents of the European North of Russia were associated to a certain extent with alterations of lipid profile whereas BMI did not influence apoE levels.

**Table 4:** Correlation coefficients of age and plasma apolipoprotein-E levels with other covariates

	**Men (n = 463)**		**Women (n = 474)**	
	Age	apoE	Age	apoE
Body mass index	0.71***	0.05	0.55***	−0.03
Total cholesterol	0.38***	0.24***	0.40***	0.15***
Triglycerides	0.33***	−0.03	0.13***	−0.10*
HDL-cholesterol	0.30***	0.04	0.42***	0.05
Glucose	0.33***	0.07	0.30***	0.03
apoE	0.26***	-	0.18***	-

Correlations are statistically significant at: * - p < 0.05; *** - p < 0.001.

## Discussion

Our results demonstrated that plasma apoE levels were not different between men and women. This fact is consistent with data from other studies [30-32]. At the same time, other authors have revealed presence of sex-related differences in apoE concentrations in humans. In some studies it has been demonstrated that apoE concentrations were higher in women, in others, apoE concentrations were higher in men [3,33,34]. It is suggested that sex hormones might cause the differences in apoE concentration between men and women [25]. Sex hormones are known to have multiple effects on lipid metabolism [39]. In particular, it has been determined that administration of estradiol to castrated male rats increased the level of apoE in lipoprotein fractions, whereas administration of testosterone to castrated female rats had the opposite effect [40]. Thus, it is possible to suppose that the hormonal status does not influence plasma apoE levels in residents of the European North of Russia or this effect is masked by other factors.

Age is supposed to be a significant factor for apoE level variation. Results of studies about age influence on apoE concentrations are conflicting. Several studies have demonstrated that apoE concentrations in blood decrease with age [41]. At the same time, there are data showing increase of apoE levels with age [33]. More detailed studies have shown that age ambiguously influences apoE concentrations in the process of maturing. Overall, apoE levels in blood decrease from birth and childhood until age 20-30 years. After age 30, apoE concentrations increase until age 60 years. The highest apoE concentrations are found between 50 and 60 years. The decrease of apoE levels was revealed after 60 years. Subjects older than 80 years have lower apoE concentrations (on 25%) than the 50-60 year-old group [23, 31, 34,]. The age dynamics of plasma apoE levels in residents of the European North of Russia revealed by us correspond to age-related changes of apolipoprotein concentrations described above. In residents of the European North of Russia, plasma levels of apoE decreased from 13 to 21 years in men and from 13 to 35 years in women and then increased in both sexes. The observed increase of apoE levels with age was probably associated with age-related changes of lipid metabolism. The increase of total cholesterol and triglycerides concentrations, the transformation of lipoprotein profile, the decrease of synthesis and number of apoB,E-receptors, the lowering of lipolitic enzymes activity and fatty acids oxidation intensity are known to be main age-related changes of lipid metabolism [42,43]. In our study, significant positive correlation between apoE and total cholesterol levels was observed in both men and women. This shows that age dynamics of plasma apoE levels in residents of the European North of Russia was primarily due to changes of total cholesterol concentration. Thus the increase of plasma apoE levels in the elder age groups in our study can be considered as a compensation of age-related changes in lipid metabolism.

In comparison with other studies, apoE levels in residents of the European North of Russia were low. In view of the variety of methods and calibrators used for apoE measurements it is difficult to compare the results received in our study with data of the literature. The apoE values reported in different studies vary widely [23]. In general, the mean values for apoE obtained by radioimmunoassay ranged from 3.6 to 6.0 mg/dl [33,44]. However, some authors reported higher results [45,46]. For enzyme-linked immunosorbent assay the average values of apoE found by different authors can be divided into two groups: between 2.0 and 4.0 mg/dl [47,48] and between 5.0 and 9.5 mg/dl [49,50]. Electroimmunoassay gives mostly the highest values, ranging from 6.7 to 11.9 mg/dl [51,52]. The reported mean concentrations of serum (plasma) apoE measured by immunoturbidimetry in control subjects are between 3.5 and 4.9 mg/dl [23,25,36].

The most suitable and interesting results were received in a large international study where a comparative estimation of apoE levels in blood among subjects from six countries of the Europe has been carried out [25,35]. According to results of this study, there is a north-south increasing gradient of apoE concentrations. The lowest apoE levels were observed in residents of Finland. ApoE concentrations (3.69 mg/dl in men and 3.56 mg/dl in women) in blood in residents of Finland (45-64 years old) were comparable to our results obtained for age-matched residents of the European North of Russia. In opinion of some authors, apoE concentration in blood is defined by the hepatic apoE production rate, not by apoE residence time in blood [20,53]. Thus, it is possible to assume that residents of the European North of Russia have a low apoE production.

The lower plasma levels of apoE in residents of the European North of Russia can be caused by features of the lipid metabolism. Lipid metabolism in residents of the North is characterized by a number of specific features due to the increased role of lipids in the energy metabolism of the human organism and specific transformations of some metabolic pathways. The alterations of energy metabolism in residents of high latitudes can be characterized as «the change-over from carbohydrate-type metabolism to the lipid one» [54]. One of the most illustrative manifestations of this phenomenon is the increase of the levels of triglycerides and VLDL in the blood of northerners. Thus low values of apoE in residents of the European North of Russia can be considered as adaptive changes providing metabolic transformation to activate lipid energy.

## Conclusion

A shift in plasma apoE levels towards lower values is characteristic for residents of the European North of Russia. Plasma apoE levels below the normal values have been observed in approximately half of the examined northerners. The low values of apoE in residents of the European North of Russia can be connected with the specific features of lipid metabolism. Thus results of this study provide additional evidence of adaptive changes of lipid metabolism in residents of the North. The received values of apoE in residents of the European North of Russia should be considered in studies of lipid metabolism in the northerners.

## Abbreviations

apoE: Apolipoprotein-E; BMI: Body mass index; VLDL: Very low density lipoproteins; HDL: High density lipoproteins

## Competing interests

The authors declare that they have no competing interests.

## Authors’ contributors

ERB and JOO were responsible for the initial conception and design of the study. NNP coordinated the blood samples collection and performed analyses. AMK contributed to the statistical analysis and interpretation data and wrote the first draft of the paper. All authors contributed to the critically revision of the article and approved the final published version to be published. All authors read and approved the final manuscript.
